# Timing of heart failure development and clinical outcomes in patients with acute myocardial infarction

**DOI:** 10.3389/fcvm.2023.1193973

**Published:** 2023-06-30

**Authors:** Hyung Yoon Kim, Kye Hun Kim, Nuri Lee, Hyukjin Park, Jae Yeong Cho, Hyun Ju Yoon, Youngkeun Ahn, Myung Ho Jeong, Jeong Gwan Cho

**Affiliations:** Department of Cardiology, Chonnam National University Medical School/Hospital, Gwangju, Republic of Korea

**Keywords:** heart failure, myocardial infarction, prognosis, acute myocardal infarct, death

## Abstract

**Background and objectives:**

To investigate the clinical relevance of the timing of heart failure (HF) development on long-term outcome in patients with acute myocardial infarction (AMI).

**Materials and methods:**

A total of 1,925 consecutive AMI patients were divided into 4 groups according to the timing of HF development; HF at admission (group I, *n* = 627), *de novo* HF during hospitalization (group II, *n* = 162), *de novo* HF after discharge (group III, *n* = 98), no HF (group IV, *n* = 1,038). Major adverse cardiac events (MACE) defined as the development of death, re-hospitalization, recurrent MI or revascularization were evaluated.

**Results:**

HF was developed in 887 patients (46.1%) after an index AMI. HF was most common at the time of admission for AMI, but the development of *de novo* HF during hospitalization or after discharge was not uncommon. MACE was developed in 619 out of 1,925 AMI patients (31.7%). MACE was highest in group I, lowest in group IV, and significantly different among groups; 275 out of 627 patients (43.9%) in group I, 64 out of 192 patients (39.5%) in group II, 36 out of 98 patients (36.7%) in group III, and 235 out of 1,038 patients (22.6%) in group IV (*P* < 0.001). MACE free survival rates at 3 years were 56% in group I, 62% in group II, 64% in group III, and 77% in group IV (*P* < 0.001).

**Conclusions:**

HF was not uncommon and can develop at any time after an index AMI, and the development of HF was associated with poor prognosis. The earlier the HF has occurred after AMI, the poorer the clinical outcome was. To initiate the guideline directed optimal medical therapy, therefore, the development of HF should be carefully monitored even after the discharge from an index AMI.

## Introduction

Heart failure (HF) is a major public health problem with over 37.7 million cases reported worldwide, and its prevalence is increasing rapidly (1). The most common cause of HF is ischemic heart disease including acute myocardial infarction (AMI), which is also growing constantly. With the advances in both optimal revascularization and medical therapy, the survival rate after AMI have improved, on the other hand, the incidence of HF associated with MI also has been increased (2, 3). The development of HF after an index MI is known to be associated with poor clinical outcomes (4, 5).

After an index AMI, HF may develop at the time of hospitalization in association with the degree of myocardial injury with a varying incidence of 14%–36% (2). As a maladaptive process for myocardial injury, so called adverse left ventricular (LV) remodeling, HF may also develop at any time of post-discharge period (6). Conflicting evidences on the incidence and temporal trend of HF after MI arises from the different definition of HF and the timing and population of HF differ among the previous reports (2, 7–9). Regardless of the type of infarct-related artery (IRA), the size of the infarcted or ischemic myocardium supplied by the IRA is a critical determinant for favorable clinical outcomes including HF prevention. In patients with chronic coronary artery diseases, routine invasive therapy for the lesions with significant myocardial ischemia failed to reduce the overall cardiovascular mortality compared to optimal medical treatment in the recent ISCHEMIA trial. However, the rapid restoration of IRA patency by percutaneous coronary intervention (PCI) and subsequent optimal medical therapy would be essential to prevent or minimize the risk of progression to HF through myocardial damage in patients with AMI. Therefore, it is important to understand the natural course of HF development or progression after MI to introduce optimal management, thereby to provide better long-term prognosis.

Contrary to the HF at the time of hospitalization for AMI, the risk of development or clinical course of *de novo* HF during post-discharge period for AMI has been poorly studied. Therefore, we investigated the post-MI clinical course for HF development and the impacts of the clinical relevance of HF development on long-term prognosis in patients with AMI.

## Materials and methods

### Study subjects and design

This is a single center retrospective and observational study, and the study protocol was approved by the institutional review board (IRB) of Chonnam National University Hospital (IRB file No. CNUH-2011-172).

We evaluated patients with clinically diagnosed for AMI at Chonnam National University Hospital (Gwangju, Korea) between November 2011 and June 2015. The diagnosis of MI was based on the criteria for a universal definition of MI: (1) when there was a rise and/or fall in cardiac biomarker values (troponin I/T or creatine kinase-MB with at least one value above the 99th percentile upper reference limit) and (2) with at least one of the following (a) symptoms of myocardial ischemia, (b) changes on the electrocardiogram (ECG) including new or presumed new significant ST-segment-T wave changes, new left bundle branch block, or pathologic Q waves in 2 contiguous leads, and (c) imaging evidence of new loss of viable myocardium or a new regional wall motion abnormality (10). Exclusion criteria included subjects with complex structural or congenital heart disease, and any clinical instability or life-threatening disease.

The diagnosis of HF was based on the following conditions, which were predominantly established from the European Society of Cardiology (ESC) guideline for AMI-associated HF: (1) cardinal manifestations of HF (such as dyspnea or fatigue), (2) rales (Killip class II or higher), (3) pulmonary edema on chest x-ray, (4) elevated level of N-terminal prohormone of brain natriuretic peptide (NT-proBNP) (11).

Patients were categorized into 4 groups according to their development of HF and their onset time of HF after an index AMI: (1) HF development at the time of admission (group I), (2) *de novo* HF during hospitalization (group II), (3) *de novo* HF after discharge (group III), and (4) no HF development during follow-up (group IV).

### Data collection

Baseline clinical, angiographic, and echocardiographic data were obtained retrospectively from medical record. Demographic and clinical data included age at diagnosis, sex, associated cardiovascular risk factors such as presence of diabetes mellitus, hypertension or smoking history, and initial presentations. Pulmonary edema on initial chest x-ray were used in the assessment of Killip class (12). Coronary angiography (CAG) and percutaneous coronary intervention (PCI) were performed according to the standard protocol (13). Decision of revascularization was made by the agreement of attending physician and interventional cardiologist. Findings of CAG were analyzed based on the ACC/AHA (American College of Cardiology/American Heart Association) classification system (14). All echocardiographic parameters including left ventricular ejection fraction (LVEF) and chamber sizes were measured according to the current recommendations for cardiac chamber quantification of American Society of Echocardiography (15, 16).

### Primary endpoint

The primary outcome of this study was a composite of major adverse cardiac events (MACE) defined as all-cause death, re-hospitalization, recurrent MI, or any revascularization.

### Statistical analysis

Data were analyzed using SPSS statistical software (version 25.0 for windows, SPSS, Inc., Chicago, IL). Categorical variables were presented as frequencies and percentages. The chi-square test or Fisher’s exact test was performed appropriately to test the difference of categorical variables among groups. In order to determine any statistical difference of continuous variables among groups, analysis of variances (ANOVA) or Kruskal-Wallis test was performed. A *post hoc* Bonferroni test for multiple comparisons was applied in order to further determine any significant differences between means among the individual groups, if any statistical differences were observed from the ANOVA or Kruskal-Wallis test. Continuous variables were presented as mean ± standard deviations. The probability of freedom from MACE of each group and survival rate were estimated by the Kaplan-Meier method and log-rank test. *P*-values <0.05 were considered as significant. Multivariable Cox proportional hazard regression analysis was performed for the adjustment of baseline characteristics.

## Results

### Baseline clinical characteristics

From November 2011 to June 2015, a total of 1,925 consecutive patients with AMI were included in this study (1,323 males, 65.7 ± 12.5 years), and HF was developed in 887 patients (46.1%) after an index AMI. HF was noted at the time of admission in 627 patients (group I: 32.6%), and *de novo* HF during hospitalization was developed in 162 patients (group II: 8.4%). Among 1,136 AMI patients who had no HF development during hospitalization, post-discharge *de novo* HF was developed in additional 98 patients (group III: 5.1%). HF was not developed in the remaining 1,038 AMI patients (group IV: 53.9%).

The clinical characteristics among the groups were statistically different in most variables, and the differences between group I and group IV were statistically significant, whereas the differences between group II and group III were not statistically significant in most variables in the post-hoc analysis.

Majority of clinical indicators, except the conventional cardiovascular risk factors, were most severe in group I and most favorable in group IV, and the values of group II and group III are allocated between the values in group I and group IV. Notably, the presence of past medical history of heart failure was not different among groups. The baseline characteristics of each group were described in detail in Table 1.

**Table 1:** Baseline clinical characteristics.

	Group I (*n* = 627)	Group II (*n* = 162)	Group III (*n* = 98)	Group IV (*n* = 1,038)	*P*-value
	Mean ± SD or *n* (%)				
Age (years)	69.7 ± 11.4	67.6 ± 12.4	67.6 ± 11.3	62.5 ± 12.4	<0.001^‡^^§^^#^
Sex, male	379 (60.4)	111 (68.5)	68 (69.4)	765 (73.7)	<0.001^§¶^
Dyspnea	163 (26.0)	22 (13.6)	7 (7.1)	50 (4.8)	<0.001*^§¶^^#^
Diagnosis					<0.001*^§¶^
STEMI	328 (52.3)	58 (35.8)	34 (34.7)	351 (33.8)	
NSTEMI	299 (47.7)	104 (64.2)	64 (65.3)	687 (66.2)	
CV Risk factors					
HTN	378 (60.3)	90 (55.6)	62 (63.3)	509 (49.0)	<0.001^‡^^§^
DM	226 (38.6)	63 (38.9)	34 (34.7)	262 (25.2)	<0.001^‡^^§^^#^
Dyslipidemia	40 (6.4)	12 (7.4)	8 (8.2)	82 (7.9)	0.702
Current smoking	180 (27.3)	44 (27.2)	27 (27.6)	408 (39.3)	<0.001^‡^^§^^#^
Previous MI	50 (8.0)	25 (15.4)	10 (10.2)	107 (10.3)	0.040*
Previous PCI	38 (6.1)	11 (6.8)	7 (7.1)	49 (4.7)	0.452
Previous CVA	60 (9.6)	11 (6.8)	8 (8.2)	52 (5.0)	0.004^§^
Previous HF	10 (1.6)	0 (0)	1 (1.0)	20 (1.9)	0.319
Killip class					<0.001*^†^^§¶^^#^
1	289 (46.1)	114 (70.4)	84 (85.7)	930 (89.6)	
2	113 (18.0)	26 (16.0)	11 (11.2)	85 (8.2)	
3	121 (19.3)	11 (6.8)	1 (1.0)	11 (1.1)	
4	104 (16.6)	11 (6.8)	2 (2.0)	12 (1.2)	

The *P*-value denotes statistical significance comparing each group. Data are listed as numbers (percentage of group), mean value. ANOVA or *χ*^2^-test for group 1 vs. group 2 vs. group 3 vs. group 4; *P*-value was calculated by one way ANOVA test and Bonferroni multiple comparisons tests for continuous variables. *P < 0.05 between group I vs. group II; ^†^group II vs. group III; ^‡^group III vs. group IV; ^§^group I vs. group IV; ^¶^group I vs. group III; ^#^group II vs. group IV. CV, cardiovascular; CVA, cerebrovascular accident; DM, diabetes mellitus; HF, heart failure; HTN, hypertension; MI, myocardial infarction; NSTEMI, non-ST-elevation MI, PCI, percutaneous coronary intervention; SD, standard deviation; STEMI, ST-elevation MI. Group I, HF at admission; Group II, *de novo* HF during hospitalization; Group III, *de novo* HF after discharge; Group IV, no HF during follow-up.

### Laboratory, echocardiography, and CAG findings

The level of CK-MB (creatine kinase MB isoenzyme), troponin I and hsCRP (high sensitivity C-reactive protein) are greatest in group I and decreased in order of later onset of HF, and lowest in group IV.

Diminished LVEF at initial echocardiography were severe in group I and II and lesser severe in order of group III, and group IV (*P* < 0.001). In addition, there was a significant difference regarding the presence of multi-vessel disease on CAG, with the higher prevalence in group I, group II, and group III, while the lowest prevalence in group IV (*P *= 0.012). Laboratory, echocardiography, and CAG findings were summarized in Table 2.

**Table 2:** Comparison of laboratory, echocardiography, angiography findings, and prescribed medications among groups.

	Group I (*n* = 627)	Group II (*n* = 162)	Group III (*n* = 98)	Group IV (*n* = 1,038)	*P*-value
	Mean ± SD or *n* (%)				
Laboratory findings					
WBC	11.95 ± 5.4	10.84 ± 3.6	9.81 ± 4.3	10.18 ± 8.3	<0.001^¶§^
Creatinine	1.29 ± 1.2	1.41 ± 1.7	1.29 ± 1.8	0.97 ± 0.9	<0.001§^#^
CK-MB	110.2 ± 165.8	97.96 ± 141.6	90.87 ± 104.7	76.76 ± 188.8	0.002^§^
Troponin I	58.98 ± 96.4	48.20 ± 69.1	52.51 ± 74.1	32.48 ± 49.1^‡^	<0.001^§^
hsCRP	2.84 ± 4.67	2.54 ± 4.82	1.37 ± 3.69	0.97 ± 2.14	<0.001^§¶^^#^
NT-proBNP (pg/dl)	7,806.1 ± 22,180.9	7,547.9 ± 10,785.5	2,234.9 ± 5,263.8	1,873.5 ± 6,736.5	<0.001^§¶^^#^
Echocardiographic parameters					
LA dimension	39.7 ± 8.0	40.1 ± 6.9	39.7 ± 5.6	37.6 ± 6.3	<0.001^‡^^§^^#^
LAVI (ml/m^2^)	43.5 ± 12.4	42.0 ± 22.0	30.3 ± 1.13	31.3 ± 9.37	0.008^§^
LVMI (g/m^2^)	116.6 ± 32.9	114.6 ± 28.5	102.9 ± 21.1	101.2 ± 24.8	0.013^§^
LVEDD (mm)	50.6 ± 6.7	52.4 ± 6.6	49.7 ± 7.0	49.2 ± 5.8	<0.001*^†^^§^^#^
LVESD (mm)	36.5 ± 7.5	38.3 ± 8.8	34.7 ± 6.2	33.0 ± 6.1	<0.001*^†^^§^^#^
IVS (mm)	9.64 ± 2.0	9.47 ± 1.7	9.82 ± 1.3	9.32 ± 1.7	0.002^§^
LVPW (mm)	9.72 ± 1.8	9.56 ± 1.6	10.0 ± 1.4	9.52 ± 1.5	0.011^‡^
LVEF (%)	47.2 ± 10.1	45.5 ± 11.7	50.1 ± 7.9	54.0 ± 8.1	<0.001^†‡^^§^^#^
LVEDV (ml)	93.2 ± 31.8	98.9 ± 36.9	86.0 ± 26.6	84.9 ± 26.5	<0.001^§^^#^
LVESV (ml)	50.3 ± 24.4	57.2 ± 31.4	43.4 ± 15.4	39.7 ± 17.7	<0.001*^†^^§^^#^
RVSP (mmHg)	40.4 ± 12.8	31.8 ± 9.6	30.8 ± 8.7	30.4 ± 6.7.	<0.001*^§¶^
Angiographic parameters					
Culprit vessel					0.043^§^
LM	28 (5.0)	3 (2.1)	4 (4.6)	20 (2.2)	
LAD	256 (45.3)	71 (49.0)	37 (42.5)	413 (45.8)	
LCX	81 (14.3)	21 (14.5)	14 (16.1)	177 (19.6)	
RCA	200 (35.4)	50 (34.5)	32 (36.8)	292 (32.4)	
Multi-vessel disease	219 (35.7)	55 (34.6)	62 (36.1)	293 (28.5)	0.012^§^
Medications					
Beta-blocker	451 (71.9)	125 (77.2)	80 (81.6)	816 (78.6)	0.010^§¶^
Calcium channel blocker	26 (4.1)	10 (6.2)	12 (12.2)	102 (9.8)	<0.001^§¶^
ACEi/ARB	456 (72.7)	128 (79.0)	82 (83.7)	857 (82.6)	<0.001^§¶^
Statin	481 (76.7)	132 (81.5)	87 (88.8)	935 (90.1)	<0.001^§¶^^#^
Warfarin	33 (5.3)	6 (3.7)	3 (3.1)	24 (2.3)	0.016^§^
Aspirin	624 (99.5)	161.1 (98.8)	98 (100)	1,032 (99.4)	0.591

The *P*-value denotes statistical significance comparing each groups. Data are listed as numbers (percentage of group), mean value. ANOVA or *χ*^2^-test for group 1 vs. group2 vs. group 3 vs. group 4; *P*-value was calculated by one way ANOVA test and Bonferroni multiple comparisons tests for continuous variables. *P < 0.05 between group I vs. group II; ^†^group II vs. group III; ^‡^group III vs. group IV; ^§^group I vs. group IV; ^¶^group I vs. group III; ^#^group II vs. group IV. ACEi, angiotensin-converting-enzyme inhibitor; ARB, angiotensin receptor blocker; EDD, end diastolic dimension; EDV, end diastolic volume; EF, ejection fraction; ESD, end systolic dimension; ESV, end systolic volume; hsCRP, high sensitivity C-reactive protein; IVS, interventricular septum; MACE, major adverse cardiac events; LA, left atrium; LAD, left anterior descending artery; LCX, left circumflex artery; LM, left main; LV, left ventricular; LVMI, left ventricular mass index; NT-proBNP, N-terminal prohormone of brain natriuretic peptide; PW, posterior wall; RCA, right coronary artery; RVSP, right ventricular systolic pressure; SD, standard deviation; WBC, white blood cell. Group I, HF at admission; Group II, *de novo* HF during hospitalization; Group III, *de novo* HF after discharge; Group IV, no HF during follow-up.

### Clinical outcomes

Clinical outcomes of the patients were summarized in Table 3. During the median 60 months (range, 0.03–93.4 months) of clinical follow-up, MACE occurred in 275 (43.9%) patients in group I, 64 (39.5%) patients in group II, 36 (36.7%) in group III, and 235 (22.6%) in group IV. MACE was significantly common in HF groups than in no HF group.

**Table 3:** Comparisons of clinical outcomes among groups.

	Group I (*n* = 627)	Group II (*n* = 162)	Group III (*n* = 98)	Group IV (*n* = 1,038)	*P*-value
	Mean ± SD or *n* (%)				
MACE	275 (43.9)	64 (39.5)	36 (36.7)	235 (22.6)	<0.001^‡^^§^^#^
All-cause death	214 (34.1)	45 (27.8)	15 (15.3)	125 (12.0)	<0.001^†^^§¶^^#^
Cardiac death	135 (21.5)	33 (20.4)	8 (8.2)	72 (6.9)	<0.001*^§¶^^#^
Re-hospitalization	47 (7.5)	13 (8.0)	10 (10.2)	42 (4.0)	0.003^‡^^§^^#^
Recurrent MI	28 (4.5)	6 (3.7)	9 (9.2)	47 (4.5)	0.177
Any revascularization	33 (5.3)	8 (4.9)	12 (12.2)	71 (6.8)	0.050^¶^

The *P*-value denotes statistical significance comparing each group. Data are listed as numbers (percentage of group), mean value. ANOVA or *χ*^2^-test for group 1 vs. group2 vs. group 3 vs. group 4; *P*-value was calculated by one way ANOVA test and Bonferroni multiple comparisons tests for continuous variables. *P < 0.05 between group I vs. group II; ^†^group II vs. group III; ^‡^group III vs. group IV; ^§^group I vs. group IV; ^¶^group I vs. group III; ^#^group II vs. group IV. MACE, major adverse cardiac events; MI, myocardial infarction; SD, standard deviation. Group I, HF at admission; Group II, *de novo* HF during hospitalization; Group III, *de novo* HF after discharge; Group IV, no HF during follow-up.

**Figure 1:**
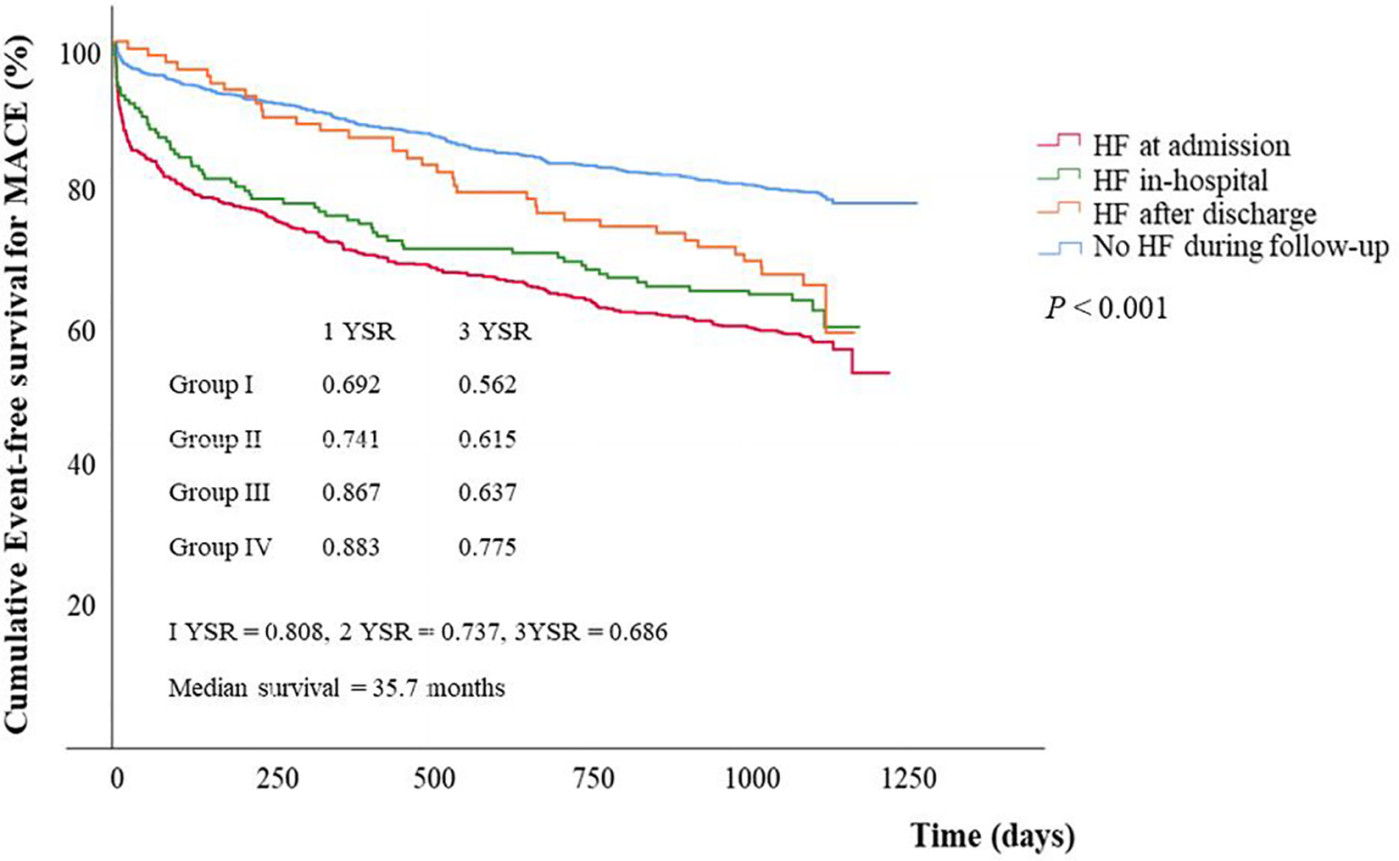
Event free survival curves for major adverse cardiac events stratified by groups. Event free survival for MACE was significantly different between group IV and any other groups (group I vs. group IV, *P* < 0.001; group II vs. group IV, *P* < 0.001; group III vs. group IV, *P *= 0.003) and between group III and group IV (*P *= 0.003), but it showed no significant difference between group I and group II (*P* = 0.247), and between group II and group III (*P *= 0.375). MACE, major adverse cardiac events; HF, heart failure; YSR, year survival rate.

Overall MACE free survival was 80.8%, 73.7%, and 68.6% at 1, 2, and 3 years, respectively. Cumulative MACE free survival at 3 years were 56.2% in group I, 61.5% in group II, 63.7% in group III, and 77.5% in group IV. Cumulative MACE free survival was statistically lower in HF groups than in no HF group (*P* < 0.001) (Figure 1). However, no statistical difference in MACE free survival was demonstrated between group I and group II (*P* = 0.247), and between group II and group III (*P* = 0.375).

All-cause mortality and cardiac death were occurred in 214 (34.1%) and 135 (21.5%) patients in group I, 45 (27.8%) and 33 (20.4%) in group II, 15 (15.3%) and 8 (8.2%) in group III, and 125 (12.0%) and 72 (6.9%) in group IV, with showing significant difference among groups (*P* < 0.001).

**Figure 2:**
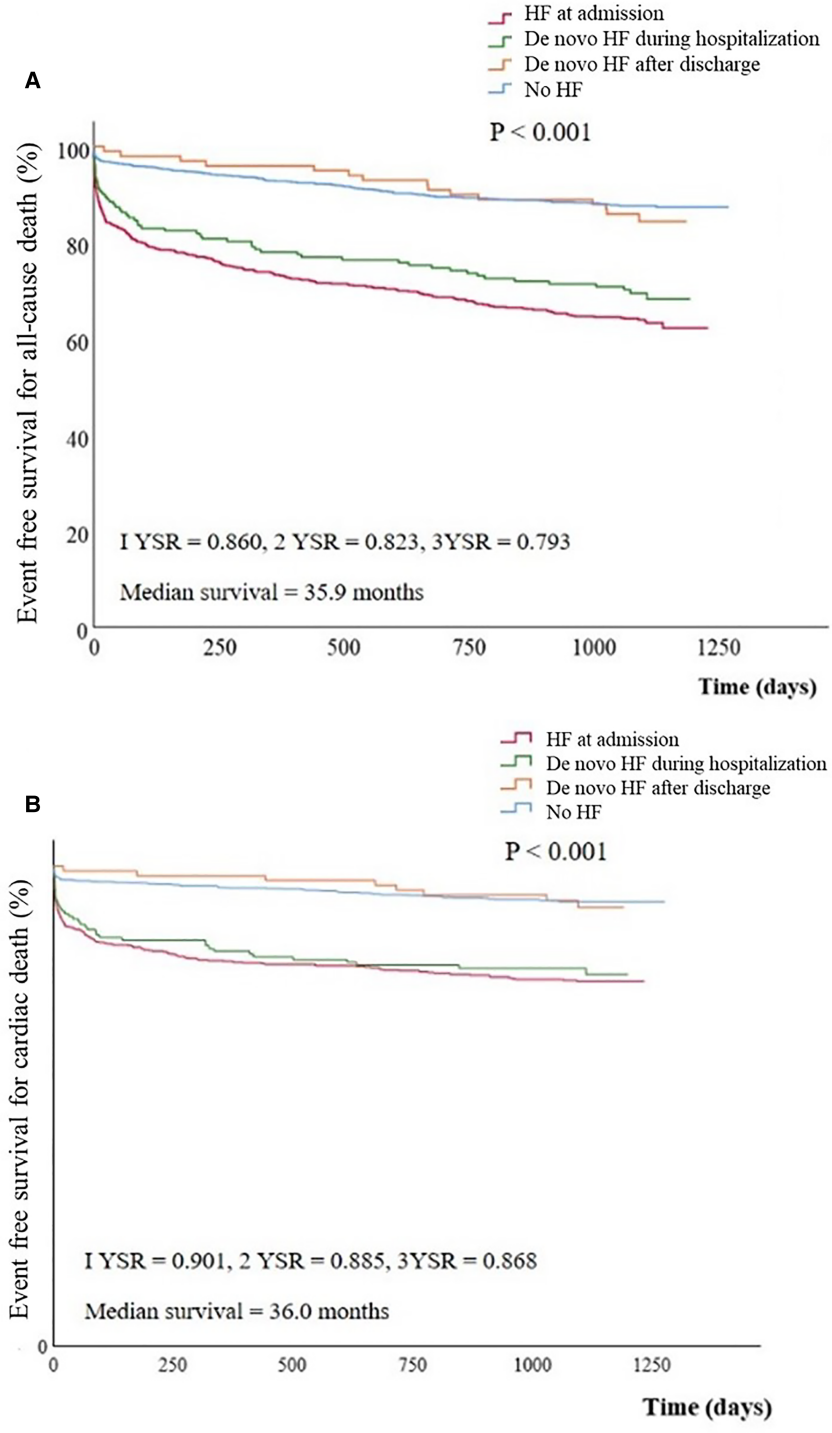
Event free survival curves for All-cause death (**A**) and cardiac death (**B**) stratified by groups. Cumulative all-cause (**A**) or cardiac death (**B**) free survival was significantly lower in group I or II than in group III or IV. (**A**) group I vs. group II, *P* = 0.100; group II vs. group III, *P* = 0.013; group III vs. group IV, *P* = 0.426; group I vs. group IV; *P* < 0.001 (**B**) group I vs. group II, *P* = 0.100; group II vs. group III, *P* = 0.007; group III vs. group IV, *P* = 0.713; group I vs. group IV, *P* < 0.001. HF, heart failure; YSR, year survival rate.

Overall cumulative survival for all-cause death and cardiac death were 86.0% and 90.1 at 1 year, 82.3% and 88.5% at 2 years, and 79.3% and 86.8% at 3 years of follow-up. Kaplan-Meier survival curves for all-cause mortality and cardiac death showed statistical differences among groups (*P* < 0.001, for all). However in the subgroup analysis, Kaplan-Meier survival curves for all-cause mortality demonstrated no statistical differences between group I and group II (*P *= 0.100) and between group III and group IV (*P *= 0.426) (Figure 2).

### Predictors of MACE

To identify independent predictors of MACE, multiple Cox regression analysis was performed, and the results were summarized in Table 4.

**Table 4:** Predictors for major adverse cardiac events.

Variables	HRs (95% CI)	
	Univariate analysis	Multivariate analysis
Age, years	1.044 (1.037–1.052)*	1.037 (1.027–1.046)*
Sex, male	1.433 (1.217–1.689)*	1.037 (0.844–1.273)
CV risk factors, <3 vs. ≥3	1.365 (1.137–1.639)*	1.438 (1.155–1.790)*
LVEF	0.957 (0.948–0.966)*	0.977 (0.970–0.985)*
Multi-vessel disease	1.264 (1.068–1.496)*	1.248 (1.023–1.522)*
Onset of HF Development, late vs. early		
Group IV	1	1
Group I	2.349 (1.973–2.796)*	1.400 (1.123–1.744)*
Group II	1.997 (1.515–2.633)*	1.332 (0.960–1.847)
Group III	1.670 (1.176–2.373)*	1.415 (0.973–2.059)
Beta-blocker Use	0.400 (0.339–0.472)*	0.640 (0.506–0.808)*
ACEi or ARB Use	0.345 (0.292–0.408)*	0.620 (0.488–0.788)*

* *P*-values <0.05. ACEi, angiotensin-converting-enzyme inhibitor; ARB, angiotensin receptor blocker; HF, heart failure; HR, hazard ratio; CI, confidence interval; CV, cardiovascular; LVEF, left ventricular ejection fraction. Group I, HF at admission; Group II, *de novo* HF during hospitalization; Group III, *de novo* HF after discharge; Group IV, no HF during follow-up.

Older age (HR 1.037, 95% CI 1.027–1.046, *P* < 0.001), number of CV risk factors ≥3 (HR 1.438, 95% CI 1.155–1.790, *P *= 0.001), LVEF (HR 0.977, 95% CI 0.970–0.985, *P* < 0.001), multi-vessel disease (HR 1.248, 95% CI 1.023–1.522, *P *= 0.029), and earlier onset of HF development after index MI (*P* = 0.017), beta-blocker use (HR 0.640, 95% CI 0.506–0.808, *P* < 0.001), and ACEi or ARB use (HR 0.620, 95% CI 0.488–0.788, *P* < 0.001) were independent predictors for MACE.

### A subgroup analysis on STEMI and NSTEMI

Among 771 STEMI patients, HF was developed in 328 patients at the time of hospitalization (group I, 42.5%), in 58 patients during hospitalization (group II, 7.5%), and in 34 patients after discharge (group III, 4.4%). HF was not developed in the remaining 351 STEMI patients (group IV: 45.5%). Whereas, HF was developed in 299 patients at the time of hospitalization (group I, 25.9%), in 104 patients during hospitalization (group II, 9.0%), and in 64 patients after discharge (group III, 5.5%) and HF was not developed in the remaining 687 patients (group IV: 59.5%) among a total of 1,154 NSTEMI patients. Kaplan-Meier survival curves for MACE showed significant difference among 4 groups in STEMI and NSTEMI patients (*P *< 0.001). However, in the results of z-test at 360, 720, and 1,080 days of STEMI patients, there were no statistical differences among the 4 groups at 1,080 days (*P* = 0.123). These findings are added in the Supplementary Figure S4. Multivariate analysis showed different results in several variables when STEMI and NSTEMI were analyzed separately. This finding is also added as a Supplementary Table S1.

## Discussion

The present study investigated the timing of HF development and its relevance to clinical outcomes in patients with AMI and demonstrated several clinically important findings. First, HF was not uncommon and can develop at any time after an index AMI including post-discharge periods. Second, regardless of the timing, the development of HF in patients with AMI was significantly associated with poor clinical outcomes. Third, the pre-discharge development of HF (at admission or during hospitalization) was associated with poorer clinical outcomes including all-cause or cardiac death in patients with AMI as compared to those with the post-discharge or no HF development. Fourth, age, LV function, number of CV risk factors, multi-vessel disease, and earlier onset of HF development were significant predictors of MACE. To initiate optimal guideline directed medical therapy for HF and improve clinical outcomes, therefore, the development of acute *de novo* HF in AMI patients who had no HF at the time of admission should be carefully monitored not only in the initial hospitalized period, but also in post-discharge period, especially in AMI patients with older age, higher level of cardiac troponin, and lower LV EF (Supplementary File).

The current universal definition of myocardial infarction and heart failure were released in 2018 and 2021, respectively (10, 17). The current definition of myocardial infarction indicates the presence of acute myocardial injury detected by abnormal cardiac biomarkers in the setting of evidence of acute myocardial ischemia. The current universal definition represents HF as a clinical syndrome with symptoms and/or signs caused by a structural and/or functional cardiac abnormality and corroborated by elevated natriuretic peptide levels and/or objective evidence of pulmonary or systemic congestion. In this study, we included patients enrolled before the release of the current guidelines, however there were no patients who were excluded from enrollment due to the changes of the definition.

HF can develop in any time after an index AMI, it occurred simultaneously with AMI in one-third of all cases, while it did not occur at all in a half of all cases in the present study. Among the baseline characteristics, it is demonstrated to be most favorable in the group IV and worst in the group I, in regards to the important clinical indicators such as older age, higher CV risk, diminished LVEF, multivessel disease. This means that patients with poor clinical indicators are more susceptible to HF development after MI. Meanwhile, previous history of HF was not significantly associated with the presence of HF or the onset of HF development after AMI.

There have been several studies reporting the outcomes and natural history of HF after AMI. A. Torabi et al. demonstrated that HF occurred in 62.7% of all subjects with MI, which was higher than our study results (18). In addition, almost one-third of patients who did not have HF at the time of discharge developed HF after discharge, which was also much higher than the result of our study. In the study, authors categorized HF groups according to early mortality, timing of onset, and persistence. Long-term prognosis of patients without HF at any time demonstrated most favorable, which is similar to our result. Another study demonstrated the increasing trend of HF development after MI over time; 5-year incidence of HF after MI was 27.6% in 1970s and rose up to 31.9% in 1990s (19). However in the recent study by Desta et al., the incidence of HF after MI declined from 46% to 28%, regardless of the onset of HF, between 1996 and 2008 (7). Limited and conflicting data may have contributed to the inconsistent results on the incidence and prognosis of HF after MI.

In the present study, event-free survival rates for MACE were significantly different according to the timing of HF occurrence. It is perceptible when considering that the baseline characteristics of groups were statistically different. Obviously, group I demonstrated the worst, and group IV demonstrated the most favorable prognosis. MACE is more likely to occur in patients who develop HF at least once, even after the discharge, compared to those who never develop HF during follow-up period. Unlikely, survival rates for all-cause mortality and cardiac death were favorable in patients who did not develop HF until discharge compared to those who developed during hospitalization. All-cause death and cardiac death were similar in patients who did not develop HF until discharge, whether HF developed or not after the index discharge. There were two different timing of delayed onset of HF after MI, in-hospitalization (group II) vs. after discharge (group III). Critical risk factors of HF including presence of pulmonary edema, higher Killip class, enlarged LV cavity size and diminished LV EF were significantly different between those two groups. The event-free survival for MACE was similar, and the overall survival for all-cause mortality and cardiac death were different between those two groups. This also supports that the earlier development of HF could be one of major risk factors of poor prognosis after MI. For better outcome, it is necessary to provide proper guideline-directed medical therapy for patients who have already experienced HF after MI, and to give close monitoring for patients who did not develop HF before their discharge as well.

There are several potential limitations in this study. First, the present study has inevitable limitations of retrospective study such as selection bias, inhomogeneity of the use of medications or percutaneous coronary intervention related parameters including reperfusion status, etc. These limitations of retrospective should be carefully considered in the interpretation of the results of this study. Second, number of group II and III were small and even unequal although relatively large number of study population, which could result in limited statistical power. Third, the diagnosis of HF was based on the symptoms and signs, which could be subjective and inaccurate indicators. However, criteria for the definition of HF also included chest x-ray and level of serum BNP to improve specificity. Fourth, the time interval of AMI onset to admission may differ for each patient, there remains a possibility of confounding bias on clinical outcomes. Further in this study, there is a possibility of immortal time bias on clinical outcomes of our study because of its retrospective design. Lastly in this study, not the timing of HF resolution but the timing of HF development was highlighted. It is likely that the temporal trends in HF after MI are associated with prognosis, not only with the development but also with its progression or improvement. In the future, the prognostic value of the temporal trends in HF after MI will be established more precisely in multicenter, prospective, larger population studies.

## Conclusions

HF development after MI is a major cause of CV morbidity and mortality. The earlier development of HF after MI is one of the independent prognostic predictors in patients with MI. To provide better outcome, careful monitoring and the guideline-directed optimal medical therapy for HF should be provided earlier in patients with MI.

## Data Availability

The raw data supporting the conclusions of this article will be made available by the authors, without undue reservation.

## Supplementary material

SUPPLEMENTARY FIGURE S1

Cumulative major adverse cardiac events free survival curves, No HF vs HF during hospitalization (*P* < 0.001).

SUPPLEMENTARY FIGURE S2

Cumulative major adverse cardiac events free survival curves, HF never vs HF ever (*P* < 0.001).

SUPPLEMENTARY FIGURE S3

A 30-day landmark analysis for event free survival curves for major adverse cardiac events (**A**) and all-cause death (**B**) stratified by groups. The MACE showed a difference between the 4 groups after 150 days. All-cause death was analyzed to have a difference between the 4 groups after 180 days. The prognostic analysis between the 4 groups showed no differences at 30 days.

SUPPLEMENTARY FIGURE S4

Event free survival curves for major adverse cardiac events (**A**) and all-cause death (**B**) stratified by groups in STEMI (**A**) and NSTEMI (**B**).

SUPPLEMENTARY TABLE S1

Predictors for major adverse cardiac events in STEMI and NSTEMI.

SUPPLEMENTARY TABLE S2

Analysis for proportional hazards for acute de novo HF in AMI patients without HF at the time of admission.
