# Supplementary material for: Timing of heart failure development and clinical outcomes in patients with acute myocardial infarction
Source: Front Cardiovasc Med. 2023 Jun 30;10:1193973. doi: 10.3389/fcvm.2023.1193973 (PMC10348359; doi:10.3389/fcvm.2023.1193973)
Supplement: Supplementary file 1 [file Presentation1.pdf]

Supplementary Figure 1. Cumulative major adverse cardiac events free survival curves, No HF vs HF during hospitalization (P < 0.001).

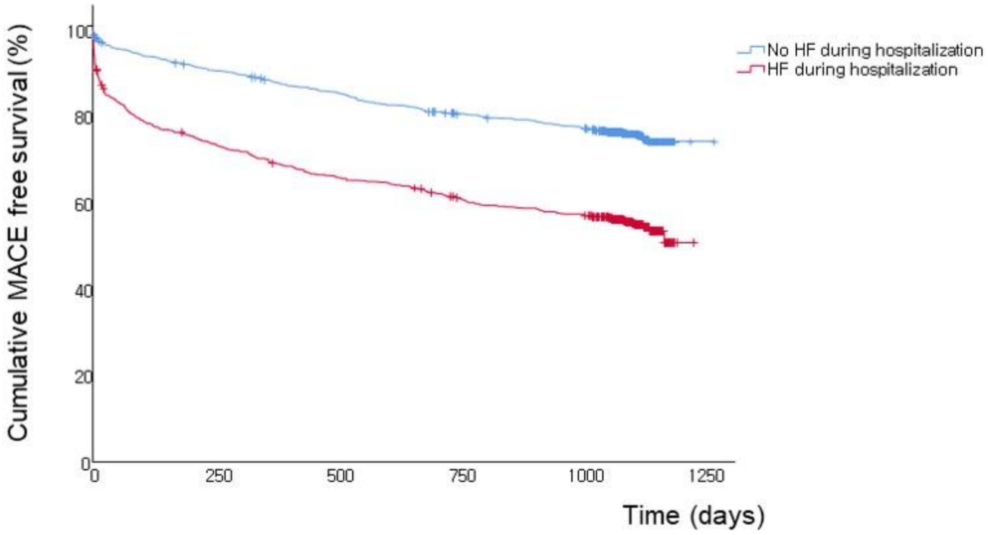

Supplementary Figure 2. Cumulative major adverse cardiac events free survival curves, HF never vs HF ever (P < 0.001).

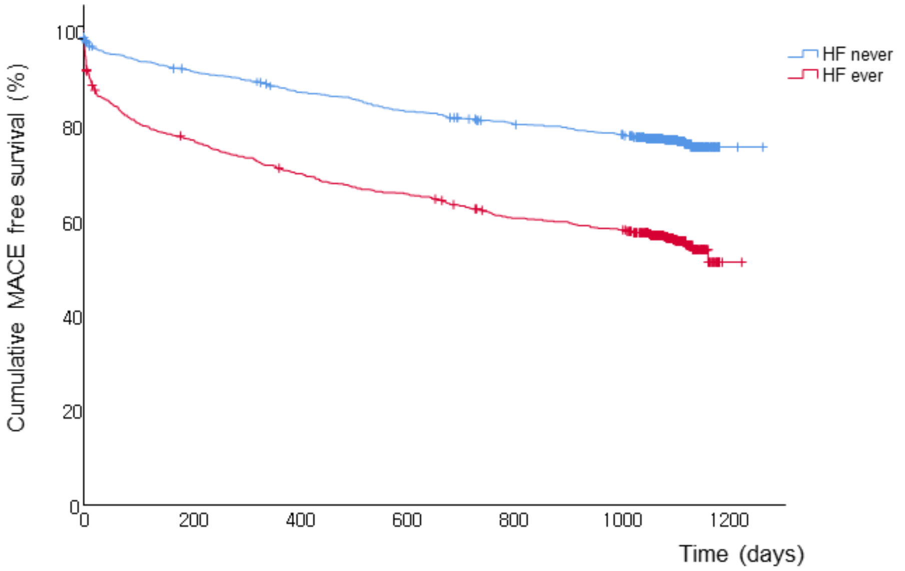

Supplementary Figure 3. A 30-day landmark analysis for event free survival curves for major adverse cardiac events (A) and all-cause death (B) stratified by groups. The MACE showed a difference between the 4 groups after 150 days. All-cause death was analyzed to have a difference between the 4 groups after 180 days. The prognostic analysis between the 4 groups showed no differences at 30 days.

(A)

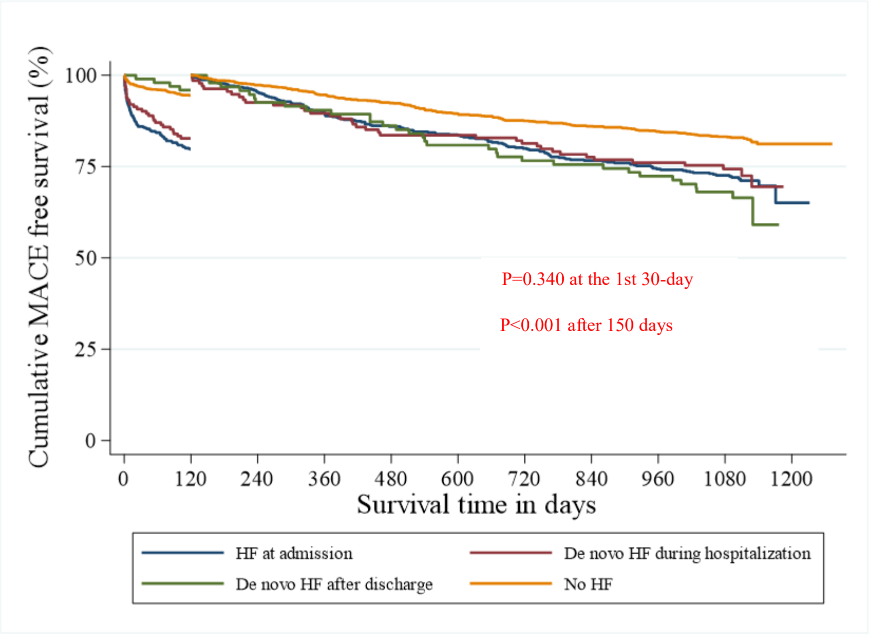

(B)

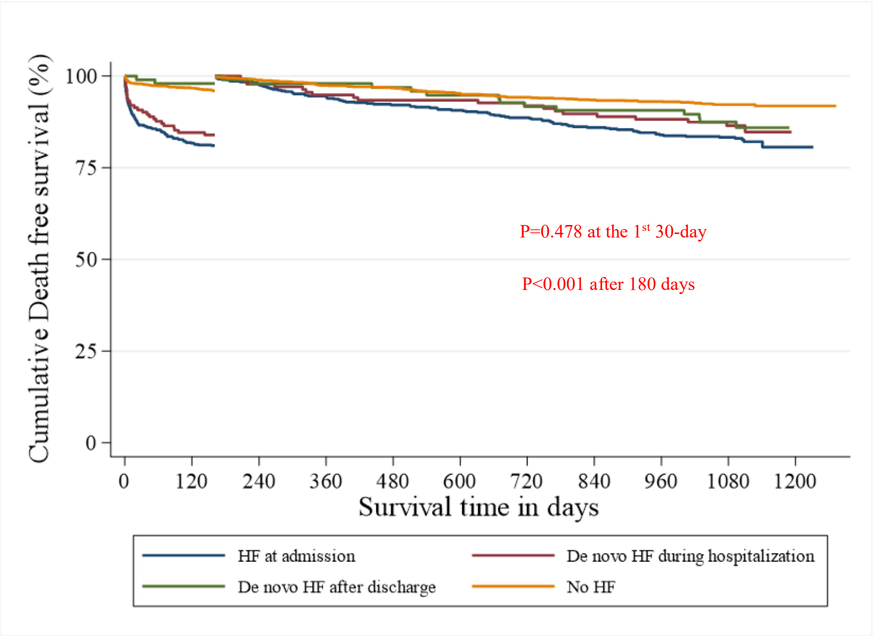

Supplementary Figure 4.

Event free survival curves for major adverse cardiac events (A) and all-cause death (B) stratified by groups in STEMI (A) and NSTEMI (B).

(A) STEMI

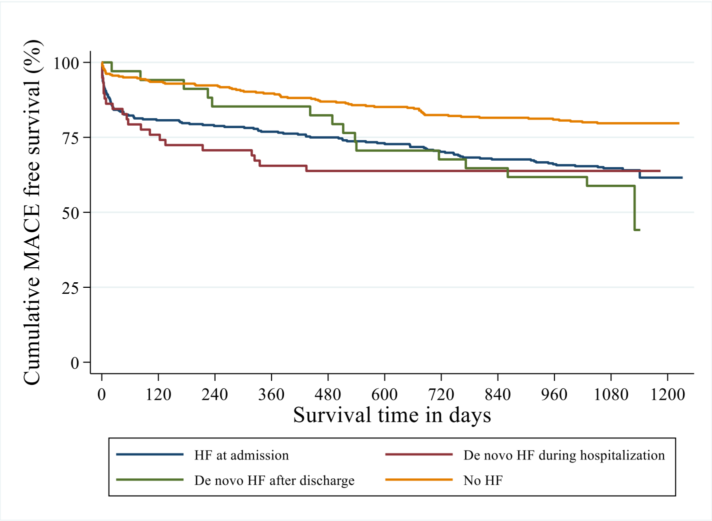

(B) NSTEMI

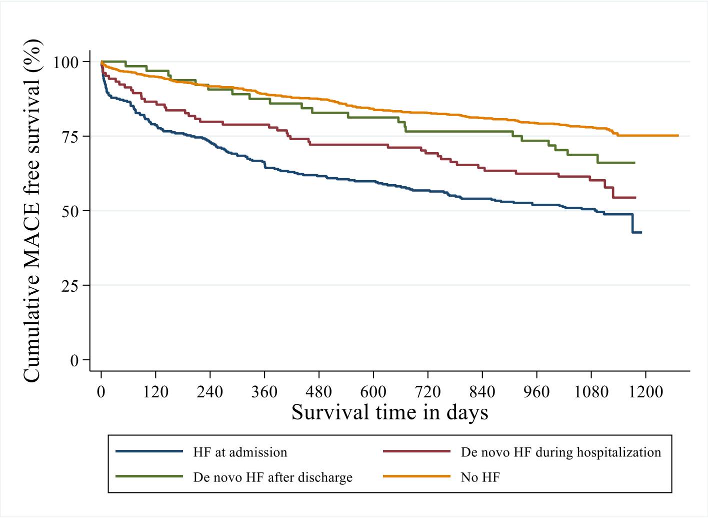

Supplementary Table 1. Predictors for major adverse cardiac events in STEMI and NSTEMI.

STEMI			NSTEMI		
	HR (95% CI)			HR (95% CI)	
	univariate	multivariate		univariate	multivariate
age	1.037 (1.025-1.049)	1.026 (1.011-1.042)	age	1.048 (1.038-1.058)	1.041 (1.029-1.053)
sex (male)	1.390 (1.047-1.846)	0.965 (0.651-1.431)	sex (male)	1.470 (1.195-1.810)	1.025 (0.804-1.307)
CV risk factors (>=3)	1.163 (0.825-1.639)	1.171 (0.743-1.847)	CV risk factors (>=3)	1.518 (1.214-1.897)	1.495 (1.158-1.931)
LVEF_M	0.972 (0.959-0.985)	0.982 (0.968-0.996)	LVEF_M	0.966 (0.957-0.974)	0.975 (0.966-0.984)
Multi-vessel disease	1.509 (1.145-1.987)	1.690 (1.196-2.387)	Multi-vessel disease	1.105 (0.888-1.376)	1.078 (0.842-1.378)
Onset of HF development			Onset of HF development		
group 4	1	1	group 4	1	1
group 1	2.009 (1.487-2.715)	1.231 (0.841-1.802)	group 1	2.868 (2.291-3.591)	1.621 (1.232-2.134)
group 2	2.160 (1.324-3.524)	1.391 (0.739-2.619)	group 2	2.072 (1.478-2.904)	1.354 (0.924-1.984)
group 3	2.324 (1.328-4.065)	2.086 (1.113-3.909)	group 3	1.484 (0.941-2.340)	1.212 (0.754-1.948)
Beta-blocker	0.255 (0.194-0.334)	0.598 (0.389-0.919)	Beta-blocker	0.625 (0.500-0.782)	0.687 (0.519-0.910)
ACEi of ARB	0.244 (0.186-0.320)	0.537 (0.356-0.809)	ACEi of ARB	0.519 (0.412-0.655)	0.643 (0.478-0.866)

Supplementary Table 2. Analysis for proportional hazards for acute de novo HF in AMI patients without HF at the time of admission.

**Variables**	**HRs (95% CI)**	
	Univariate	Multivariate
**Age, years**	1.038 (1.027 – 1.049)*	1.045 (1.026-1.063)*
**Sex**	1.317 (1.012 – 1.712)*	0.708 (0.621-1.381)
**CV risk factors, < 3 vs ≥ 3**	1.192 (0.889 – 1.600)	0.982 (0.604-1.636)
**Troponin I**	1.004 (1.002 – 1.005)*	1.003 (1.001-1.006)*
**NT-proBNP**	1.000 (1.000 – 1.000)	1.000 (1.000-1.000)
**LVEF by echocardiography**	0.954 (0.944 – 0.964)*	0.950 (0.936-0.964)*
**Multi-vessel disease**	1.309 (1.012 – 1.691)*	0.762 (0.524-1.110)
**Diagnosis (STEMI or NSTEMI)**	0.930 (0.722 – 1.200)*	0.652 (0.417-1.021)

HF: heart failure, AMI: acute myocardial infarction, CV: cardiovascular, NT -proBNP: N terminal pro B type natriuretic peptide, LVEF: left ventricular ejection fraction, STEMI: ST elevation myocardial infarction, NSTEMI: non-ST elevation myocardial infarction. * indicates p value < 0.05.
